# Showing Behaviour in One Hundred and One Dogs: Gazing, Breed and Cephalic Index

**DOI:** 10.3390/ani16050760

**Published:** 2026-03-01

**Authors:** Samuele Commauda, Veronica Maglieri, Emanuela Prato-Previde, Elisabetta Palagi

**Affiliations:** 1 Ethology Unit, Department of Biology, University of Pisa, Via Volta 6, 56126 Pisa, Italy; s.commauda@gmail.com (S.C.); veronica.maglieri@biologia.unipi.it (V.M.); 2 Department of Pathophysiology and Transplantation, University of Milan, 20122 Milan, Italy; emanuela.pratoprevide@unimi.it

**Keywords:** dog–human communication, referential signalling, gaze alternation, out-of-reach paradigm, hidden object task, companion dogs, effects of life experience

## Abstract

Dogs exhibit sophisticated interspecific communication skills, including the use of visual signals to indicate the location of inaccessible resources, known as showing behaviour. Previous studies have investigated factors such as age and training, but the effects of breed and cranial morphology remain unclear. Here, we tested a uniquely large sample of 101 pet dogs from 43 different breeds, using a standardized out-of-reach/hidden object task to assess three key visual behaviours: gaze at the owner, gaze at the reward, and gaze alternation between owner and reward. Dogs were tested in familiar environments without pre-training, and owners were instructed to remain passive to avoid unintentional cues. Our results confirm the importance of gaze alternation and gazing at the reward as central components of showing behaviour, particularly when both owner and reward were present. Contrary to expectations, we found no effect of breed or Cephalic Index on these behavioural patterns, suggesting that life experiences rather than artificial selection can influence visual communicative strategies in this specific context. The exceptionally large and diversified sample of this study provides unprecedented insight into the consistency of visual signalling across dog breeds.

## Simple Summary

Dogs are very good at communicating with humans. One important way they do this is by using visual signals, such as looking back and forth between a person and an object they cannot reach, to show where a desired resource is located. This behaviour is known as showing behaviour. Previous research has focused on the role of age and training, but it is still unclear whether dog breed or head shape influence this type of communication. In this study, we tested 101 pet dogs from 43 different breeds using a simple task in which food was hidden or placed out of reach. We recorded how often dogs looked at their owner, at the food, and alternated their gaze between the two. Our results show that gaze alternation and looking at the reward are key elements of showing behaviour. Importantly, these behaviours did not differ across breeds or head shapes, suggesting that everyday experience with humans, rather than selective breeding, plays a major role in shaping dogs’ visual communication.

## 1. Introduction

Several studies have been conducted to shed light on the nature and extent of the mutual understanding between dogs (*Canis lupus familiaris*) and humans [1]. Research on dog social cognition has explored different aspects of dog–human communication, showing that dogs possess the ability to detect and respond to a various array of human signals [2,3,4,5,6,7,8] and are skilful in emitting various signals to influence their human partners’ behaviours [9]. Both these abilities are considered a pivotal step toward achieving a high level of cooperativeness [10,11,12].

Dogs are particularly responsive to visual cues provided by humans such as various pointing gestures and body/head orientation [3,13]. They are also able to follow the direction of the human gaze and perceive/decode human attentional states [14,15]. Moreover, dogs are sensitive and respond to the human gaze and show human-directed gazing as a communicative tool during various types of interactions [16,17].

Gazing at others, and in particular alternating gaze between a social partner and a specific target (e.g., a desired resource), is considered a way of initiating communication by attracting and directing the partner’s attention toward a location or object [18]. These gazing patterns play a significant role in social interactions and are even employed as a form of referential communication [19].

Previous works indicate that gazing at humans is influenced by ontogenetic factors such as age [20] and life experiences [21,22,23]. Moreover, artificial selection has shaped dogs’ visual communicative abilities toward humans, leading some breeds to be more visually oriented than others [24]. According to previous evidence, cooperative breeds (e.g., Border Collie, Standard Poodle) and dogs with a long history of artificial selection (e.g., Labrador Retriever, German Shepherd) rely more on gazing behaviour than ancient breeds (e.g., Afghan Hound, Saluki) and wolfdogs during visually based tasks [24,25,26,27,28,29]. In addition, skull conformation, as quantified by the Cephalic Index, may further influence gazing behaviour by affecting orbital orientation and visual field overlap. For instance, brachycephalic dogs (e.g., English Bulldog, French Bulldog) appear to establish eye contact with their owners more rapidly [30].

Gazing behaviour constitutes the core of the so-called “showing behaviour”, defined by Miklósi and colleagues [31] as an interspecific behaviour in which dogs display an array of visual signals aimed at indicating to a naïve human the presence of a hidden object or food. This behaviour is considered a communicative act comprising an attention-getting component, aimed at attracting attention from a social partner, and a directional component, related to an external target [31,32].

The common visual-based task that is used to demonstrate showing behaviour is the “out-of-reach/hidden object task” [19,31]. Such a task is commonly employed to evaluate if/how dogs provide information to their owners about the location of an inaccessible hidden reward that dogs cannot reach by themselves. Trials assessing whether dogs engage in showing behaviour are typically accompanied by two control conditions: one in which the dog is alone with the reward hidden out of reach, and another in which the owner is present, but no reward is available. These conditions are introduced to assess the effects of the mere presence of the food or the owner on the dog’s behaviour, respectively [31,32,33].

Despite the variability in protocols, settings and sample sizes, studies indicate three visual patterns (gazing at the owner, gazing at the reward location, and gaze alternation between the owner and the reward location) as key indicators of showing behaviour [19,32,34,35,36]. Some studies concentrated on the effect of the training the dogs underwent on differences in showing behaviour (pet versus guide dogs [36]; pet versus dogs for animal-assisted interventions [35]). One study focussed on the effect of age in the development of showing behaviour (puppies versus adults [32]). Overall, neither training nor age had any impact on the ability to use visual strategies during these tasks.

Despite the selection operated by humans on different communicative traits in dogs, to our knowledge, no studies evaluated the effect of breeds on the different communicative strategies employed during the out-of-reach/hidden object task. This study aimed at filling the gap by applying a strict protocol on 101 dogs belonging to 43 different breeds to further explore the visual tactics adopted by dogs in showing behaviour. This large sample size allows taking into account dog sex, age, breed lineage and Cephalic Index (CI), considering the effect of individual variability. Dogs were tested in their familiar context to prevent possible distress due to novel environments to which different subjects can react in different ways [37]. To prevent any bias due to unintentional association and incidental learning, no dog was exposed to any pre-training session. Since human behaviour can strongly affect the performance of dogs during cognitive and communicative tasks [33,38,39], we asked the owner not to gaze at/talk to/touch the dog to avoid any unintentional reinforcement on the dog behaviour. For the same reason, the owner was totally unaware not only about the presence of food but also about its possible location [32].

In this study, we aimed to expand the knowledge of the showing behaviour, not just replicating previous findings on a larger sample size, but testing the role of artificial selection on this peculiar referential inter-specific visual communication.

If gaze alternation, gazing at the inaccessible reward (food, in this study), and gazing at the owner are reliable components of showing behaviour in dogs, these responses should be most frequent when the communicative partner (the owner) and the referent (the reward) are simultaneously present (“Food + Owner”), compared to conditions in which only the reward or only the owner is available. Moreover, since brachycephalic and cooperative dog breeds with a long history of selection for close interaction with humans tend to rely more strongly on visual communicative strategies with their owners [24,26,39,40], we expected these groups to show higher levels of gaze alternation, owner-directed gazing, and looking towards the reward location.

## 2. Materials and Methods

### 2.1. Ethic Statement

The present study has been authorized by the Animal Welfare Authority of the University of Pisa (approval n. 14/2022). Moreover, it was authorized by the Committee on Bioethics of the University of Pisa (Protocol n. 97310, 1 August 2022). Owner’s data were entered in an anonymous form (an alphanumerical code has been uniquely assigned to each subject). Owners were asked to sign an informed consent allowing data collection and videorecording.

### 2.2. Subjects

The dogs were recruited through personal contacts, advertisements on social media, dog’s training facilities, and by word of mouth in the provinces of Livorno, Pisa, Lucca, and La Spezia (Italy). A total of 113 purebred dogs were tested. We considered several features while selecting a dog (e.g., sex, age, breed, size, Cephalic Index) to make our sample as representative as possible. The criteria for selection included choosing purebred dogs that were at least six months old and lived as indoor pets in a household, and never experienced mistreatment. Dogs under six months of age were excluded to minimize the effect of the early social developmental period, typically occurring between three to four months of age [41]. Adolescence in dogs has been associated with transient changes in responsiveness and increased behavioural variability toward caregivers, but these effects appear to be context-dependent and strongly shaped by individual attachment quality, rather than representing a uniform shift in communicative strategies [42]. The age cut-off was set to exclude dogs in the early socialization period, which is more consistently linked to the development of human-directed communication [42]. It has been shown that gazing toward humans increases with age regardless of breed [20], and that, at approximately six months of age, dogs display showing behaviour in the presence of out-of-reach hidden food [32].

Information on the dogs’ life history was collected via owner reports. Only dogs whose owners indicated no history of abuse or mistreatment were included. While this cannot entirely rule out unreported adverse experiences, this approach is standard in studies of companion dogs living in household environments. All dogs had lived continuously with their current owners since adoption and approximately 75% of the dogs were adopted before three months of age (Table S1). Table S1 reports the distribution of dogs according to age at adoption, illustrating that most individuals were adopted between two and three months of age.

Out of the 113 dogs initially tested, 12 were excluded from the analysis: eight dogs (5 females, 3 males) exhibited fearfulness, and four dogs (4 females) were excluded due to set-up problems. Thus, 101 dogs were included in the analyses (51 females and 50 males; mean AGE= 4.61 ± 0.3 SE; ageMIN= 0.5 year; ageMAX = 13 years).

### 2.3. Training for the Experimenters

To familiarize ourselves with the procedure, the experimenters (SC, VM, and an assistant) underwent an initial training during which they simulated the actions and gestures they would execute during the actual test trials. The goal was to achieve a consistent level of behavioural similarity between the experimenters (EXPs) during the testing process. Two female Golden Retrievers were tested. Data from these subjects were not included in the subsequent analyses.

### 2.4. Lineage

The dogs were categorized into seven breed groups based on the most recent genetic grouping information, as outlined in the study conducted by Dutrow and colleagues [43]. These breed groups include Herding, Ancient–Spitz, Retriever, Pointing–Water dogs, Sighthound, Toy, and Mastiff–Terrier. Table S2 reports the distribution of dogs across breeds and lineages, highlighting the uneven representation across groups.

### 2.5. Cephalic Index

Georgevsky and colleagues [44] warned against the use of a priori head shape groups (brachycephalic, mesocephalic, dolichocephalic) or breed average Cephalic Index (CI) values (proposed by dogs’ Kennel clubs) due to the substantial variability in Cephalic Index within a breed, as highlighted by Bognár and colleagues [30].

Hence, we quantified the CI for each dog, enabling its use as a continuous variable in subsequent analyses. Following previous studies [30,45,46,47], dog heads were photographed from a dorsal perspective, maintaining the snout parallel to the ground. This approach enabled the measurement of both skull length and width. A fabric strap with a rectangular benchmark (2.5 cm, 5 cm) was fastened around the widest part of the dog’s head, connecting the two zygomatic arches at their broadest point. To facilitate later measurement of the distance from the occipital crest to the frontmost part of the nose, one of the EXPs placed their finger marked with a pen on the dog occipital crest, otherwise difficult to detect in the picture (Figure S1). We took at least three photographs for each dog (PANASONIC^®^ VXF990). We utilized ImageJ (version 1.54m), an open-source software designed for the processing and analysis of scientific images, to measure the photographs captured of each dog. Following image scaling facilitated by the benchmark, we computed the dimensions of each dog’s head, specifically measuring its length and width in each picture. The length was measured from the marked fingertip to the tip of the snout, whereas the width was measured from one zygomatic arch to the other. Finally, we calculated the CI from these three measurements by taking the mean of the lengths and the mean of the widths, along with their respective standard errors (SEs).

### 2.6. Task Procedure

The tests were carried out from March 2022 to February 2023 and were conducted in the familiar places where the dogs lived. The test was preceded by a familiarization period (15–20 min) to habituate the dogs to the EXPs’ presence. During this phase, the EXPs explained to the owners each stage of the experiment, outlining the specific rules they needed to adhere to. The EXPs emphasized the critical instruction of avoiding gazing at/interacting with the dog throughout the entire procedure to avoid any unintentional communication (e.g., mere eye contact), which could potentially influence the dog behaviour, thus compromising the validity of the test.

**Figure 1:**
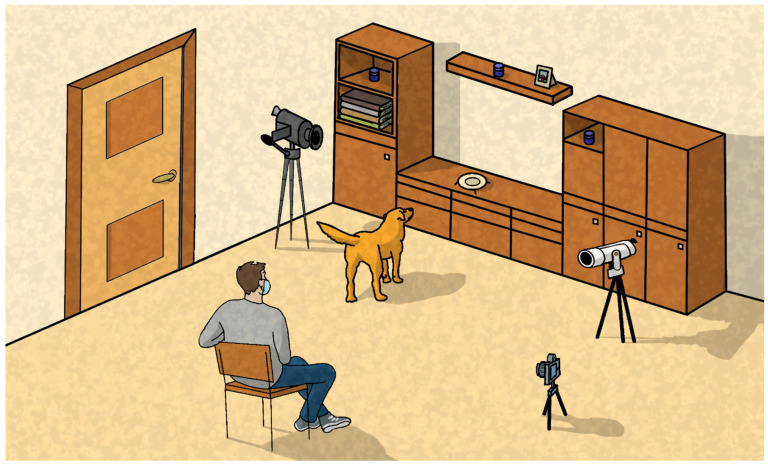
Design of the testing setting. All trials took place in a room inside the dogs’ and owners’ homes. Three cameras caught the entire scene while three small, opaque blue containers were positioned in elevated, mutually remote locations out of the dogs’ reach. Both the owner and the researcher wore normal light-blue surgical masks, and the owner had a seat. Drawing by Samuele Commauda.

Three opaque circular blue pots (3 × 4.5 cm)—blue being a colour perceived by dogs [48,49]—were placed on tables, furniture or shelves present in the room. The location selected allowed dogs to see and smell without any possibility of reaching the pot on their own. The pots were placed at a minimum distance of 1.5 metres from each other at a minimum height of one metre (Figure 1). In all cases, the owner present during testing was the person primarily responsible for the dog’s daily care and interaction. In households with multiple caregivers, the individual most consistently involved in the dog’s daily management and interaction was selected to participate in the test.

During the test, both the owner and a single experimenter (EXP) wore a standard light-blue surgical mask, thus minimizing any potential communication bias arising from unintentional lip movements. Given that the dogs had been regularly exposed to their owners wearing surgical masks during the COVID-19 pandemic, all the dogs were accustomed to them (Figure 1).

Three wide-angle cameras (Canon^®^ PowerShot SX710 HS, Canon Inc., Tokyo, Japan—Nikon^®^ Coolpix P500, Nikon Corporation, Tokyo, Japan—PANASONIC^®^ VXF990, Panasonic Corporation, Osaka, Japan, accuracy 0.02 s) were placed inside the room to maximize the probability to spot all the behaviours of the dog.

### 2.7. Procedure

The task included three different trials.

(1) Owner + Food trial—the EXP and the dog were alone in the room. The EXP held a piece of food and showed it to the dog that could sniff it. Then, the EXP put the food inside one of the three pots, making sure the dog was looking at the action. The EXP left the room and let the owner (unaware whether food was present or not) enter the room. The owner sat on a chair and ignored the dog for 1 min.

(2) Owner-only trial—the EXP and the dog were alone in the room. The EXP had no food in their clean hands and showed them to the dog, who was free to sniff them. The EXP left the room and let the owner (unaware whether food was present or not) enter the room. The owner sat on a chair and ignored the dog for 1 min.

(3) Food-only trial—the EXP and the dog were alone in the room.

The EXP held a piece of food and showed it to the dog that could sniff it. Then, the EXP put the food inside one of the three pots, making sure the dog was looking at the action. The EXP left the room and the dog remained alone in the room for 1 min.

All the trials were randomized using an online free application (https://play.google.com/store/apps/details?id=com.appsnblue.smartdraw&hl=it, accessed on 25 July 2023). At the beginning of each trial, the EXP opened the pots and showed each of them to the dog. Just for the first trial involving the reward (Owner + Food or Food-only), a single piece of food was directly offered to the dog from the baited container to allow the dog to associate the pot with the possible presence of the reward. Providing the single piece of food also served to boost the dog’s motivation to engage with the baited container or signal the presence of food to the owner. Small pieces of chicken sausage were the main food used. In a few cases (N = 10), a different meat-based treat was provided by the owner, based on the dog’s individual preference. All dogs showed clear interest in the food, as indicated by approaching, sniffing, and attention to the experimenter during food presentation. At the end of each trial in which the owner was present, the EXP asked the owner about whether and where the food might have been hidden based on the dog’s behaviours, and the information was recorded on a table-sheet.

### 2.8. Video Analysis

Videos were analyzed using the programme PotPlayer (Kakao Corp., Seongnam-si, South Korea). We clustered and coded dogs’ behaviours into four categories.

Gazing at the owner (seconds)—the dog turned/lifted its head and gazed at the owner’s face. This behaviour was not coded during the Food-only trial.

Gazing at the full pot (seconds)—the dog turned/lifted its head and gazed at the pot with a piece of food inside. This behaviour was not coded during the Owner-only trial.

Gaze alternation (bouts)—one alternation was coded as switching directly from gazing at the owner’s face to gazing at the full pot within two seconds (or vice versa) [33,34,50]. In the Owner-only trial, one gaze alternation was coded as switching directly from gazing at the owner’s face to gazing at the empty pot corresponding to the full pot in the same position in the Owner + Food condition within two seconds (or vice versa). This behaviour was not coded during the Food-only trial.

Self-directed behaviours—mouth-licking (seconds) used as a conservative behavioural indicator of a mild anxiety-related state.

SC and VM were the principal coders of videos. To check for inter-coder reliability, two field assistants independently analyzed 30 videos as blind coders (about 10%). They were unaware about the aim of the study and were denied access to a portion of the video that showed the setting preparation, i.e., unaware about the presence/absence of food and the location of the pots. The coders noted all the behaviours performed by the dogs related to the pots, the owner, and self-directed actions. Then, 10 videos were selected to assess the inter-coder reliability across all the four coders. We calculated the Cohen’s K for each of the six possible coder dyads, and it always exceeded the 0.9 score.

### 2.9. Statistical Analysis

Five main models were used to investigate distinct components of showing behaviour, each addressing a specific outcome variable and a predefined set of condition contrasts. Generalized Linear Mixed Models (GLMMs; glmmTMB R-package, version 1.1.14 [51]; R Core Team, 2020) were used to examine the role of visual behaviours in the showing task. All response variables, with the exception of those modelled as binomial outcomes (Models 2a and 5), consisted of discrete count data (e.g., number of events or duration expressed as integer seconds). These variables were therefore analyzed using a negative binomial error distribution (nbinom2) to account for overdispersion. Models with binary response variables were analyzed using a binomial error distribution. An overview of all models, including outcome variables and condition contrasts, is provided in Table S3.

Model 1—Gazing at the owner (seconds). The response variable was the amount of time (seconds) dogs spent gazing at the owner. The fixed factors were the condition (Owner + Food/Owner-only), the sex and the age of the dog, breed lineage, the Cephalic Index, and the trial number (1/2/3). No collinearity between the fixed factor was found (VIFmax = 1.29; VIFmin = 1.02).

Model 2a—Probability of gazing at the full pot (binomial). This model was set to understand if the dog discriminated between the pots with food and the empty ones. To build this model, we produced a spreadsheet in which, for each trial where food was present, we recorded whether the dog looked at the pot containing food or at one (or both) of the other empty pots. This resulted in four rows per dog: two for the “Owner + Food” and two for the “Food-only” condition. The response variable was the presence/absence of at least one gaze at one of the pots. The fixed factors were the condition (Owner + Food/Food-only), the type of the pot (full/empty), the sex and the age of the dog, breed lineage, the Cephalic Index, and the trial number (1/2/3). No collinearity between the fixed factor was found (VIFmax = 2.73; VIFmin = 1.02).

Model 2b—Gazing at the full pot (seconds). The response variable was the amount of time (seconds) dogs spent in gazing at the full pot. The fixed factors were the condition (Owner + Food/Food-only), the sex and the age of the dog, the breed lineage, the Cephalic Index, and the trial number (1/2/3). No collinearity between the fixed factor was found (VIFmax = 2.65; VIFmin = 1.01).

Model 3—Gaze alternation (bouts). The response variable was the number of gaze alternation events, while the fixed factors were the condition (Owner + Food/Owner-only), the sex and the age of the dog, breed lineage, the Cephalic Index, and the trial number (1/2/3). No collinearity between the fixed factors was found (VIFmax = 1.67; VIFmin = 1.03). In the Owner-only condition, we calculated the gaze alternations exhibited by the dogs when they alternated the gaze at the owner and the empty pot corresponding to the full pot in the same position in the Owner + Food condition.

Model 4—Owners’ match (binomial). The response variable was the presence/absence of owners’ matching in the Food + Owner condition. The fixed factors were the number of gaze alternations, the time spent by dogs in gazing at the full pot, the time spent by dogs moving around, the time spent in attention-getting behaviours (e.g., barking, touching), the sex and the age of the dog, the breed lineage, the Cephalic Index, and the trial number (1/2/3). No collinearity between the fixed factor was found (VIFmax = 1.56; VIFmin = 1.05).

Model 5—Mouth-licking (bouts). The response variable was the number of mouth-licking events. The fixed factors were the condition (Owner + Food/Food-only/Owner-only), the sex and the age of the dog, the genetics, the Cephalic Index, the trial number (1/2/3), and the interaction between the condition and the breed lineage (condition*CI), and the interaction between the condition and the lineage (condition*lineage). No collinearity between the fixed factor was found (VIFmax = 2.66; VIFmin = 1.04).

We used the Likelihood Ratio Test to test the significance of the full model (LRT, Anova with argument test “Chisq” [52]), by comparing the full model (from model 1 to 4) against a control one comprising only the random factors (identity of the dog, identity of the owner) and the fixed factors trial order (1/2/3), sex and age. In model 5, we compared the full model against a null model comprising only the random factors (identity of the dog, identity of the owner). We chose to include the identity of the owner as a random factor because one owner can correspond to more than one dog. We also chose to include the trial order as a control fixed factor because it is possible that, as the tests progress, the dog could have a loss of motivation, increased frustration, or some form of learning due to the sequential presentation of the three trials. Previous works took into account these factors when conducting the analysis (e.g., [34,53,54,55]).

Then, by using the R-function “Anova” (car package, version 3.1-5 [56]), we calculated the *p*-values for the individual predictors. To exclude the occurrence of collinearity among fixed factors, we examined the variance inflation factors (Performance package, version 0.12.2 [57]). We performed all pairwise comparisons for the levels of the multilevel factor with the Tukey test by using the R package emmeans (v1.5.3) [58].

## 3. Results

### 3.1. Model 1

Response variable: gazing at the owner (seconds). The full model built to investigate which factors influenced the time the dog spent gazing at the owner did not differ from the control model including only the random and the control factors (trial order, sex, age) (LRT: χ^2^ = 0.790, df = 3, *p* = 0.852). Estimated marginal means for gazing at the owner were very similar between the Owner-only condition (mean = 3.45 s, 95% CI [2.28, 5.21]) and the Owner + Food condition (mean = 3.67 s, 95% CI [2.46, 5.46]), with largely overlapping confidence intervals.

### 3.2. Model 2a

**Figure 2:**
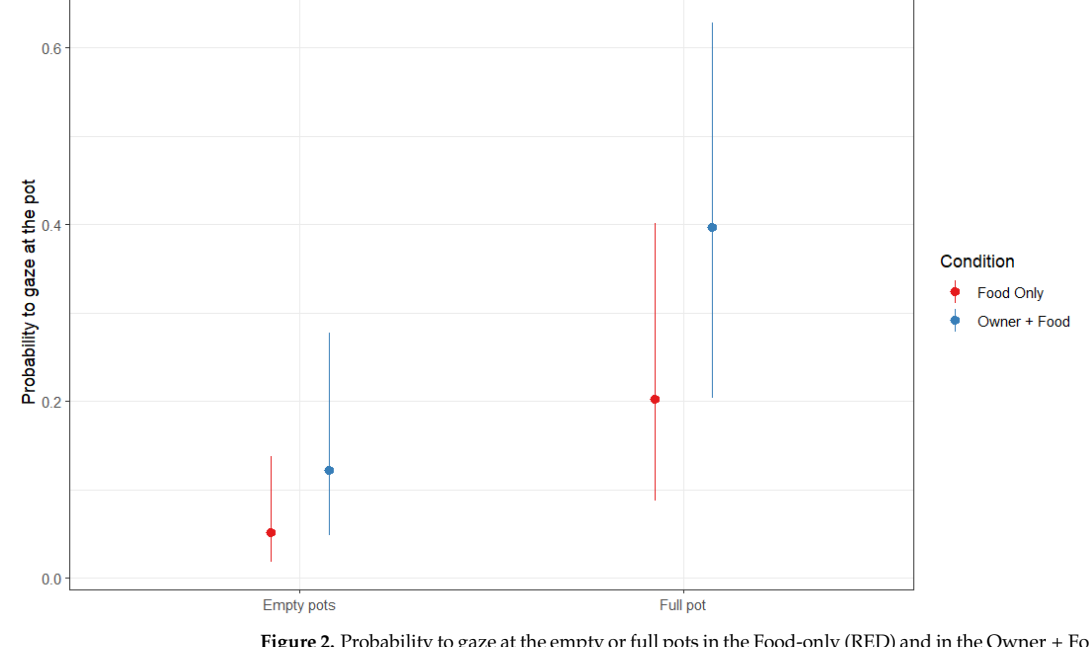
Probability to gaze at the empty or full pots in the Food-only (RED) and in the Owner + Food condition (BLUE).

Response variable: probability of gazing at the full pot (binomial). The full model built to investigate which factors influenced the occurrence of gazing at the full pot significantly differed from the control model including only the random and the control factors (trial order, sex, age) (LRT: χ^2^ = 54.90, df = 9, *p* < 0.001). The fixed factor condition had an effect on the response variable, as dogs looked preferentially at the full pot in the “Owner + Food” than in “Owner-only” condition (Figure 2, Table 1).

### 3.3. Model 2b

**Figure 3:**
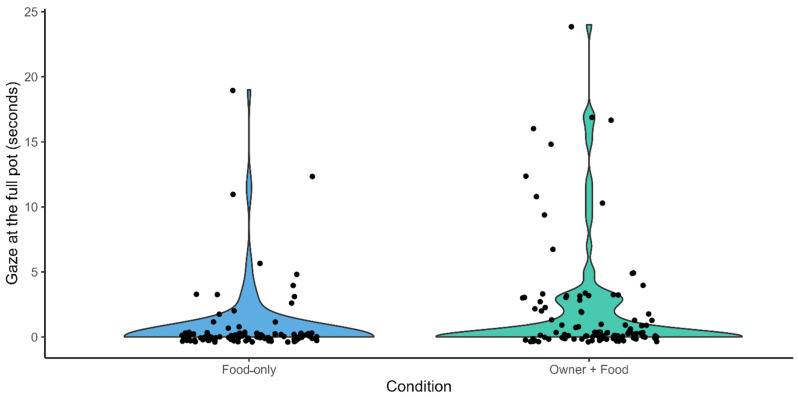
Violin plots showing the time spent by dogs in gazing at the full pot in the Food-only (BLUE) and in the Owner + Food condition (GREEN). Each dots represent a trial session.

Response variable: time spent at gazing at the full pot (seconds). The full model built to investigate which factors influenced the occurrence of gazing at the full pot significantly differed from the control model including only the random and the control factors (trial order, sex, age) (LRT: χ^2^ = 19.051, df = 8, *p* = 0.015). The fixed factor condition had an effect on the response variable, as dogs spent more time gazing at the full pot in the “Owner + Food” than in the “Food-only” condition (Figure 3, Table 1).

### 3.4. Model 3

**Figure 4:**
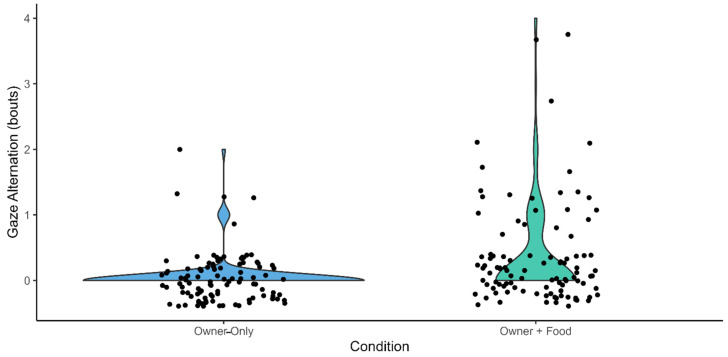
Violin plots showing the number of gaze alternation events in the Owner-only (BLUE) and in the Owner + Food condition (GREEN). Each dots represent a trial session.

Response variable: gaze alternation (bouts). The full model built to investigate which factors influenced the number of gaze alternations significantly differed from the control model including only the random and the control factors (trial order, sex, age) (Likelihood Ratio Test: χ^2^ = 18.704, df = 3, *p* < 0.001). The fixed factor “condition” had an effect on the response variable, as dogs exhibited a higher number of gaze alternation in the “Owner + Food” than in “Owner-only” condition (Figure 4, Table 1).

### 3.5. Model 4

Response variable: owners’ matching (binomial). The full model built to investigate which factors influenced owners’ ability to accurately determine whether the food was present or not did not differ from the null model including only the random factors (LRT: χ^2^ = 20.553, df = 13, *p* = 0.082). Predicted probabilities of correctly inferring the presence of food were similarly low when dogs did not gaze at the full pot (approximately 13%) and when they did (approximately 15%). Confidence intervals were wide and strongly overlapping (95% CI [0.04, 0.38] vs. [0.05, 0.39]), indicating a very small effect size.

### 3.6. Model 5

Response variable: mouth-licking (bouts). The full model built to investigate which factors influenced the time dog spent in mouth-licking did not differ from the control model including only the random and the control factors (trial order, sex, age) (LRT: χ^2^ = 32.693, df = 23, *p* = 0.087). Estimated marginal means differed numerically across conditions (FO: mean = 0.15, 95% CI [0.08, 0.27]; OO: mean = 0.26, 95% CI [0.16, 0.44]; SH: mean = 0.53, 95% CI [0.35, 0.80]). However, confidence intervals were broad and partially overlapping, and the full model did not differ significantly from the control model, indicating substantial variability and the absence of a robust condition effect. Additionally, we also recorded the yawning events that were very rare (N yawns = 17 emitted by just 11 dogs).

## 4. Discussion

This study explores dog–human-directed communication, and in particular gazing behaviour, in a standardized out-of-reach/hidden object paradigm, testing a large sample (N = 101) of pet dogs from several different breed groups. The dogs’ behaviour was assessed considering the dogs’ breed lineage and Cephalic Index (CI), which is a way to classify dogs based on their individual cranial anatomic features [44], an approach never attempted in previous studies to our knowledge. The analysis on mouth-licking (i.e., a proxy for anxiety) suggests that dogs do not seem to experience anxiety across the different trials. Also, yawning behaviour, another proxy for anxiety, was extremely rare in our dogs, supporting the data emerged from the mouth-licking analysis.

Before discussing the results in detail, it is important to frame them within the specific communicative context in which the study was conducted. Dogs were all pet tested indoors, in their familiar environment, with a passive, masked, and nonresponsive owner. This design choice was adopted to minimize unintentional cueing and to standardize human behaviour across trials. At the same time, it represents a particular interactional setting that may reduce certain forms of owner-directed communication. Accordingly, the following interpretations primarily apply to this sample and this experimental protocol without any kind of overgeneralization.

The results partially supported our initial predictions, which were based on the existing literature on the out-of-reach/hidden object task [19,32]. Dogs exhibited more gaze alternations when both the owner and food were present than when the owner was present, but the designated pot was empty. This pattern aligns with prior studies on dogs’ gaze alternation and its role in “showing behaviour” [31,32,33,34,35,36,38,47,59,60,61] and supports its interpretation as a referential communicative behaviour aimed at indicating a target location to a human partner [33,62].

In line with our predictions, dogs gazed more at the full pot in the presence of the owner, confirming previous evidence that gazing at the reward location is a behaviour used to signal the presence of food to the owner [31,32,34,35,50]. On the other hand, when the owners were not present in the room, dogs spent less time in gazing at the full pot, revealing that they perceived the role played by their owners in obtaining the reward.

Contrary to our prediction based on previous findings [19,32,34], we did not observe any difference in gazing at the owner when both the reward and the owner were present, compared to when the owner was present, but the designated pot was empty. This finding suggests that, in our test situation, this behaviour was not employed as a strategy to capture the owners’ attention or await a possible human reaction. The studies by Savalli and colleagues [19,34] included a training phase in which dogs learned that, although the experimenter placed the food on the shelf, it was the owner who would ultimately provide it. Thus, the dogs repeatedly received food from their owner prior to testing. In the procedure used by Prato-Previde and colleagues [32], the experimenter showed the dogs that the bowls could contain food, placed the pots on the floor, and allowed them to eat what was inside before the test began.

It is possible that our results on human-directed gazing depended, to some extent, on methodological differences, as our dogs never received food by their owners before the beginning of the test. In addition, the similarity we observed in gazing at the owner across conditions may also be attributable to the owner’s complete lack of reaction. The owner was trained to ignore the dog throughout the entire procedure and wore a surgical mask covering half of the face to prevent any form of positive reinforcement.

This could also explain the difficulty of the owners in precisely figuring out whether there was food or not at the end of the trial, further highlighting how a passive and nonresponsive human partner may limit the expression and interpretation of certain communicative behaviours in this context.

Dogs are sensitive and responsive to a human’s attentional state, and even slight communicative cues or lack of communication from humans can modulate their behaviour [13,39,63]. Finally, depending on the situation, the dogs’ gazing behaviour may have different underlying motives, such as affection and comfort seeking, seeking help or for information [16,64,65].

Contrary to our expectations, we did not observe any variations in the use of gaze across the seven different lineages of dogs tested in the present study. The lack of variability across breeds is consistent with previous evidence highlighting the role of life experiences in gazing behaviour [26,64,65,66,67]. It suggests that shared experiences and everyday environmental factors may have a stronger influence than lineage or breed, although subtle breed-related effects cannot be ruled out. All dogs in our study were companion animals living with their families. They had not received any formal training but had extensive everyday experience interacting with humans. In the “impossible task”, wolves, dingoes and less selected dog breeds gaze less at humans compared to more selected and human-oriented dogs [20,24,26,27,68,69]. The difference between our findings and the findings of this task could depend, at least on some extent, on the different nature of the two tasks. The absence of lineage effects should also be interpreted in light of the uneven distribution of dogs across breeds and lineages (Table S2), which reflects the diversity of the sample but also results in limited representation for some groups. In particular, the most represented lineage included 23 individuals (Mastiff–Terrier lineage), whereas the least represented lineages included 10 dogs each (Ancient–Spitz and Toy lineages), potentially limiting the statistical power to detect subtle lineage-related differences. During the recruitment of the subjects, we could not find scent hound dogs reared as pets, because of the typical use of these breeds during hunting/scent activities with humans and reared in kennel structures. So, to further confirm our findings, future research should include dogs reared as pets and dogs with different degrees of artificial selection such as mongrels or free-ranging dogs.

Similar results emerged when looking at the Cephalic Index (CI). We initially expected that individuals with the highest CI values (i.e., strongly brachycephalic groups) might exhibit a higher level of gazing behaviours, as suggested by previous studies [30,70,71,72]. In contrast to Ujfalussy and colleagues [73], who compared brachycephalic and mesocephalic breeds, we found no evidence of an association between CI and gazing behaviour, possibly due to the variability of this index across breeds and individuals. Our findings regarding breed lineages and CI further support the hypothesis that variability arising from artificial selection is unlikely to affect dogs’ performance in the showing task. The interest of brachycephalic dogs in human faces may, in part, reflect humans’ heightened attention to their faces, owing to the paedomorphic traits characteristic of the so-called infant schema [74,75]. The results reveal that, despite individual differences, dogs adaptively use visual cues to convey information to humans, illustrating the interplay between inherent traits and life experiences in guiding showing behaviour.

## 5. Conclusions

Our findings indicate that gaze alternation and attention to the reward are key components of dogs’ showing behaviour, particularly in the presence of a human partner. The results support the view that these behaviours act as referential communicative signals, guiding human attention to a relevant target. At the same time, the absence of increased gazing at the owner across conditions suggests that human-directed gazing may be strongly modulated by contextual and methodological factors, including prior learning history and the human’s attentional and communicative availability. Importantly, within this controlled experimental setting, we found no evidence that breed lineage or Cephalic Index influenced dogs’ gazing strategies. This lack of variation across lineages and cranial morphologies can suggest a homogeneity in visual communicative behaviour among owned pet dogs tested in this context and, possibly, the effect of everyday life experiences with humans that may override differences introduced through artificial selection. In addition, measuring the CI for each subject could have emphasized the importance of the individual differences in gazing behaviour, as already reported in a previous study analyzing the effect of individuality, rather than breed, in shaping personality traits [76]. Together, these results underline the flexibility of dog–human communication and underline the individual variability in shaping dogs’ visual signalling, providing new insight into the robustness of showing behaviour across the domestic dog population.

## Figures and Tables

**Table 1:** Results from the models. Lineage refers to “breed lineage”.

**Model 2a** **—** **Response variable: gazing at one of the pots (N = 404)**			
Tested variables			
**Variable**	**χ^2^**	**df**	***p***
Condition	10.777	1	**0.001**
Type of pot	24.820	1	**<0.001**
Cephalic Index	1.050	1	0.306
Lineage	12.395	6	0.054
Control variables			
Sex	0.198	1	0.657
Age	4.270	1	0.039
Trial order	1.443	1	0.230
Variance of the random factors: ID_dog_ = 1.037 ± 1.019, ID_owner_ = 6.935 × 10^−9^ ± 8.328 × 10^−5^			
**Model 2b** **—** **Response variable: time spent gazing at the full pot (N = 202)**			
Tested variables			
**Variable**	**χ^2^**	**df**	***p***
Condition	12.591	1	**<0.001**
Cephalic Index	1.556	1	0.212
Lineage	7.867	6	0.248
Control variables			
Sex	0.001	1	0.974
Age	0.083	1	0.774
Trial order	1.102	1	0.294
Variance of the random factors: ID_dog_ = 1.051 ± 1.025, ID_owner_ = 6.087 × 10^−8^ ± 0.00025			
**Model 3** **—** **Response variable: number of gaze alternation (N = 202)**			
Tested variables			
**Variable**	**χ^2^**	**df**	***p***
Condition	13.642	1	**<0.001**
Cephalic Index	0.332	1	0.565
Lineage	0.214	6	0.643
Control variables			
Sex	0.014	1	0.905
Age	2.236	1	0.135
Trial order	4.396	1	0.036
Variance of the random factors: ID_dog_ = 1.711 ± 1.308, ID_owner_ = 1.019 × 10^−5^ ± 0.003			

## Data Availability

The original contributions presented in this study are included in the article/Supplementary Material. Further inquiries can be directed to the corresponding authors.

## Supplementary Materials

The following supporting information can be downloaded at: https://www.mdpi.com/article/10.3390/ani16050760/s1, Table S1. List of the dogs tested and their age at adoption; Table S2. Summary table reporting, for each breed, its corresponding lineage and the number of individuals tested; Table S3. Summary of all statistical models used to analyse showing behaviour, indicating for each model the outcome variable, error distribution, and condition contrasts; Table S4. Estimates and SE of the significant models.
