# Supplementary material for: Showing Behaviour in One Hundred and One Dogs: Gazing, Breed and Cephalic Index
Source: Animals (Basel). 2026 Mar 1;16(5):760. doi: 10.3390/ani16050760 (PMC12985302; doi:10.3390/ani16050760)
Supplement: Supplementary file 1 [file animals-16-00760-s001.zip › animals-4089820-supplementary.pdf]

Table S1. List of the dogs tested and their age at adoption.

Breed Group	Breed	N	Sex	Age
Herding (n=17)	Australian Cattle Dog	2	F=1, M=1	5.5±2.5
	Australian Shepherd	5	F=3, M=2	5.3±0.97
	Border Collie	3	F=2, M=1	2.42±1.31
	Czechoslovakian Wolfdog	1	M	12 ± 0.0
	Collie	2	F=2	5.5±1.5
	German Shepherd	4	F=2, M=2	5±1.35
Terrier (n=4)	Jack Russell	4	F=1, M=3	7.54±2.14
Pointing (n=4)	Italian Pointer	2	F=1, M=1	4.5±0.5
	Brittany	1	M	3±0.0
	Dachshund	1	M	2±0.0
Ancient-Spitz (n=9)	Alaskan Malamute	1	M	1.17±0.0
	Podengo	1	F	3.5±0.0
	Siberian Husky	5	F=4, M=1	3.9±1.64
	Shiba Inu	2	F=1, M=1	4±1
Retriever-Water dogs (n=22)	Cavalier King	1	F	7±0.0
	Cocker Spaniel	3	F=1, M=2	4±1.15
	Golden Retriever	9	F=7, M=2	4.56±0.71
	Labrador Retriever	2	F=1, M=1	8.75±4.25
	Lagotto Romagnolo	3	F=1, M=2	0.92±0.05
	Papillon	1	F	2±0.0
	Poodle	2	M=2	2.5±0.5
	Springer Spaniel	1	M	0.67±0.0
Sighthound (n=16)	Borzoi	1	F	1.17±0.0
	Galgo	8	F=4, M=4	6.58±1.16
	Greyhound	5	F=1, M=4	2.55±0.82
	Italian Greyhound	1	M	4,5±0.0
	Whippet	1	F	8±0.0
Toy (n=10)	Chihuahua	5	F=1, M=4	4.7±1.75
	Lhasa Apso	1	M	1±0.0

	Maltese	1	M	2±0.0
	Pug	2	F=1, M=1	0.79±0.04
	Shih tzu	1	M	9±0.0
Mastiff (n=19)	American Staffordshire Terrier	1	F	3±0.0
	Bernese Mountain Dog	1	F	0.5±0.0
	Boxer	3	F=2, M=1	6,28±0.86
	French Bulldog	5	F=2, M=3	5±1.14
	Bull terrier	1	M	8.5±0.0
	Corso	1	M	9±0.0
	Dobermann	1	F	5.5±0.0
	English Bulldog	3	F=3	5±1.5
	Hovawart	1	F	5±0.0
	Leonberger	1	M	1.83±0.0
	Saint Bernard	1	F	5±0.0

Table S2. Summary table reporting, for each breed, its corresponding lineage and the number of individuals tested.

Breed Group	Breed	N	Sex	Age
Herding (n=17)	Australian Cattle Dog	2	F=1, M=1	5.5±2.5
	Australian Shepherd	5	F=3, M=2	5.3±0.97
	Border Collie	3	F=2, M=1	2.42±1.31
	Czechoslovakian Wolfdog	1	M	12 ± 0.0
	Collie	2	F=2	5.5±1.5
	German Shepherd	4	F=2, M=2	5±1.35
Ancient- Spitz (n=10)	Alaskan Malamute	1	M	1.17±0.0
	Borzoi	1	F	1.17±0.0
	Podengo	1	F	3.5±0.0
	Siberian Husky	5	F=4, M=1	3.9±1.64
	Shiba Inu	2	F=1, M=1	4±1
Retriever (n=11)	Golden Retriever	9	F=7, M=2	4.56±0.71

	Labrador Retriever	2	F=1, M=1	8.75±4.25
Pointing-Water Dogs(n=15)	Brittany	1	M	3±0.0
	Cavalier King	1	F	7±0.0
	Cocker Spaniel	3	F=1, M=2	4±1.15
	Dachshund	1	M	2±0.0
	Italian Pointer	2	F=1, M=1	4.5±0.5
	Lagotto Romagnolo	3	F=1, M=2	0.92±0.05
	Papillon	1	F	2±0.0
	Poodle	2	M=2	2.5±0.5
	Springer Spaniel	1	M	0.67±0.0
Sighthound (n=15)	Galgo	8	F=4, M=4	6.58±1.16
	Greyhound	5	F=1, M=4	2.55±0.82
	Italian Greyhound	1	M	4,5±0.0
	Whippet	1	F	8±0.0
Toy (n=10)	Chihuahua	5	F=1, M=4	4.7±1.75
	Lhasa Apso	1	M	1±0.0
	Maltese	1	M	2±0.0
	Pug	2	F=1, M=1	0.79±0.04
	Shih tzu	1	M	9±0.0
Mastiff - Terrier (n=23)	American Staffordshire Terrier	1	F	3±0.0
	Bernese Mountain Dog	1	F	0.5±0.0
	Boxer	3	F=2, M=1	6,28±0.86
	French Bulldog	5	F=2, M=3	5±1.14
	Bull terrier	1	M	8.5±0.0
	Corso	1	M	9±0.0
	Dobermann	1	F	5.5±0.0
	English Bulldog	3	F=3	5±1.5
	Jack Russell	4	F=1, M=3	7.54±2.14
	Hovawart	1	F	5±0.0
	Leonberger	1	M	1.83±0.0
	Saint Bernard	1	F	5±0.0

Table S3. Summary of all statistical models used to analyse showing behaviour, indicating for each model the outcome variable, error distribution, and condition contrasts

**Model**	**Outcome variable**	**Error distribution**	**Condition contrasts**
**Model 1**	Gazing at the owner (s)	Negative binomial	OWNER+FOOD *vs* OWNER ONLY
**Model 2a**	Probability of gazing at pots	Binomial	OWNER+FOOD *vs* FOOD ONLY × full *vs* empty pot
**Model 2b**	Gazing at the baited pot (s)	Negative binomial	OWNER+FOOD *vs* FOOD ONLY
**Model 3**	Gaze alternation (counts)	Negative binomial	OWNER+FOOD *vs* OWNER ONLY
**Model 4**	Owners' matching	Binomial	OWNER+FOOD
**Model 5**	Mouth licking (counts)	Negative binomial	OWNER+FOOD *vs* OWNER ONLY *vs* FOOD ONLY

Table S4. Estimates and SE of the significant models.

**Model 2a – Response variable: gazing at one of the pots (N=404)**		
Tested variables		
**Variable**	**Estimate**	**SE**
(Intercept)	-0.759	1.196
Condition	0.950	0.290
Type of pot	1.548	0.311
Cephalic index	-0.018	0.018
Lineage Ancient-Spitz	0.978	0.657
Lineage Retriever	-2.300	0.937
Lineage Pointing-Water dogs	-0.378	0.636
Lineage Sighthound	-0.285	0.632
Lineage Toy	0.579	0.818
Lineage Mastiff-Terrier	0.260	0.625
Control variables		
Sex	0.166	0.373
Age	-0.135	0.065
Trial order	-0.233	0.194
**Model 2b – Response variable: time spent gazing at the full pot (N=202)**		

Tested variables		
**Variable**	**Estimate**	**SE**
(Intercept)	1.156	1.607
Condition	1.509	0.425
Cephalic index	-0.030	0.024
Lineage $_{\text{Ancient-Spitz}}$	0.647	0.872
Lineage $_{\text{Retriever}}$	-2.155	1.034
Lineage $_{\text{Pointing-Water dogs}}$	0.444	0.812
Lineage $_{\text{Sighthound}}$	-0.145	0.845
Lineage $_{\text{Toy}}$	0.585	1.100
Lineage $_{\text{Mastiff-Terrier}}$	0.658	0.826
Control variables		
Sex	-0.016	0.492
Age	-0.026	0.090
Trial order	-0.287	0.273
**Model 3 – Response variable: number of gaze alternation (N=202)**		
Tested variables		
**Variable**	**Estimate**	**SE**
(Intercept)	-0.967	1.602
Condition	1.663	0.448
Cephalic index	-0.020	0.023
Lineage $_{\text{Ancient-Spitz}}$	0.127	0.885
Lineage $_{\text{Retriever}}$	-0.630	1.036
Lineage $_{\text{Pointing-Water dogs}}$	-0.077	0.859
Lineage $_{\text{Sighthound}}$	-0.153	0.850
Lineage $_{\text{Toy}}$	0.395	1.062
Lineage $_{\text{Mastiff-Terrier}}$	0.480	0.859
Control variables		
Sex	0.060	0.500
Age	-0.121	0.086
Trial order	-0.475	0.885
